# Observations on the Reciprocal Propagation of Disease between the Thoracic and Abdominal Viscera

**Published:** 1828-09

**Authors:** 


WESTERN JOURNAL

OF THE

### MEDICAL & PHYSICAL SCIENCES.

SEPTEMBER, 1828.

### *ESSAYS AND CASES.*

Art. I.—*Observations on the reciprocal Morbid Influences of the Abdominal and Thoracic Viscera.*—By the Editor.

Most diseases are seated in one or more of the great cavities—cranial thoracic, abdominal and pelvic. It rarely happens that any malady extends to the whole; but examples of the propagation of a morbid state from the organs of one cavity to the organs of another, are far from uncommon. I propose briefly to inquire into the modes in which diseases of the abdominal or portal viscera may raise diseases in the thoracic and *vice versa.*

I. Of the various ways in which the portal viscera may affect, unfavorably, the thoracic.

1. By Sympathy. I restrict the term sympathy to the influence which one organ exercises over another, through the instrumentality of the nervous system. How that system establishes associate and sympathetic actions, between organs remote from each other, we can no more explain, than we can tell why the same system makes us acquainted with the existence and properties of surrounding bodies. As without its aid, in the latter mode, we should be ignorant of what passes about us; so without its aid, in the former case, our organs would, in a great degree, be unacquainted with each others condition. They would not be combined into a physiological unit; but remain insulated, except as far as they depended for the necessary nutriment on a common fountain. They would be like ununited provinces, scattered along the banks of a common river, from which they might all obtain supplies, and grow and flourish, according to the volume of its waters, while they were utterly unconscious of each others existence. Now it is the nature of the nervous function, to make the well-being of one organ, in some degree, a cause of the well-being of other organs; and the ill-being of one organ, a cause of the ill-being of other organs—sometimes one, sometimes more.

The question here arises, whether the organ diseased being given, we can determine *a priori*, what organ or organs will suffer through sympathy? I answer that, in general, we cannot. We might suppose that the viscus, most intimately associated with the affected organ, would be the first and greatest sufferer; but we do not find such, invariably, to be the fact; and many reasons might be assigned, why, if that assumption be theoretically, true, it may be often practically false. Thus, in the first place, there are other modes of propagation of disease, besides the one we are now considering, which may often modify or countervail it; and secondly, if an organ, congenitally or casually, happen to be in a bad condition, when disease is awakened in some other organ, the former may become the sympathizing sufferer, however remotely situated, or slightly connected, in its functions, with the latter.

But to return to the object in view, we repeat that disorder in the functions, of one or more of the abdominal viscera frequently carries disorder into the functions of the thoracic viscera, through the medium of the nervous tissue. The sub and super diaphragmatic organs are supplied by nerves, which have the most intimate anatomical relations. The pulmonary and cardiac plexuses are both formed by detachments from the pneumogastric nerves, united with others from the intercostal ganglions; and the splanchnic, have a similar composition, with an augmented proportion of ganglionic branches. Hence the nerves of the heart and lungs originate contiguous to those of the liver and stomach, and are, of course, liable to be morbidly affected by the morbid states of the latter.

2. Excess of digestive function may contribute to the production of thoracic disease, by generating a plethoric state of the circulatory system: Thus palpitations and hypertrophies or hœmoptysis may sometimes depend, in part, though remotely, on excessive functional excitement in the digestive organs.

3. When inflammation, in one of the abdominal viscera, extends its influence to the heart and brain, and induces, in their functions, the change which leads to symptomatic fever, the heart is disordered by sympathy. In this state of increased energy and action, it may raise an inflammation in some of the thoracic tissues, which would, thus, become diseased through its intervention, presenting us with a different mode of propagation from either of those already mentioned.

4. The veins of the stomach may absorb foreign matters, which being carried directly to the heart and lungs, may act as irritants, and induce, or predispose to disease.

5. The digestive function of the stomach, bowels, liver and mesenteric glands, being disordered, chyle of an unhealthy quality must necessarily be prepared, which, being transmitted to the thoracic viscera, cannot fail to pervert their vital properties, and contribute to the production of a morbid state.

6. When the liver fails in the secretion of bile, and its elements are left in the blood, they prove injurious to the health of the heart and lungs, in common with the other organs of the body. As the bile performs an important office in the bowels, we cannot refer it to the class of purely excrementitious fluids, like the urine and perspiration; but still there are numerous facts which demonstrate, that its elements are developed in the blood, and must be eliminated from it. As it respects the blood vessels, they are excrementitious; and to the lungs they seem especially prejudicial. They probably irritate that organ into a kind of vicarious excretory action. Between the liver and lungs, there is this analogy that they are both transmitting organs of venous blood;—

one for the abdominal viscera, the other for the whole body; and at the same time, they exert upon it a species of depuratory action. An interrupted excretion of bile from the ducts of the liver, is known to be followed by the return of that fluid to the blood, where it plays nearly the same mischievous part with the retained elements. It is worthy of remark, that the influence of the biliary elements is exerted largely upon the serous membranes, producing dropsical effusions, and hence, in part, the frequency of hydrothorax and hydropericardium in hepatic disorders.

7. Enlargement of the liver prevents the full and easy descent of the diaphragm, and thus embarrasses the lungs in their operations.

8, And lastly. The muscles of respiration are subservient to several offices of the abdominal viscera; such, for example, as vomiting and fæcal evacuation; and, therefore, it is necessary to give to these organs the power of exciting those muscles. This is effected through the medium of the nervous system, and is, indeed, a species of sympathy. In certain kinds of abdominal irritation, the muscles of respiration are thrown into spasmodic action; as we see in a paroxysm of asthma from acid or bile in the stomach.

It seldom happens, indeed never, that the morbid influence of the abdominal over the thoracic functions is exercised in any single mode here pointed out. They are of necessity combined. Thus, in dyspepsia, the lungs and heart are affected through the nervous system, that is sympathetically, and at the same time, such unhealthy chyle may be sent to those organs as must contribute to the production of their derivative maladies. And, thus, when the liver is inflamed, the lungs and the respiratory muscles may be injured through the nervous tissue, while, simultaneously, the secretions of bile being, partly or wholly, suspended, the lungs or their membranes may suffer greatly from that cause, in the manner pointed out.

As to the relative influence of the different modes which I have assigned, it would be difficult—or rather impossible—to speak with precision. The principal seem to me to be the three following, which are influential in the order of enumeration.—1. Nervous connexion. 2. Suspended or perverted hepatic secretion. 3. Morbid chylification.

II. Of the morbid influence of the thoracic viscera, upon the abdominal.

The organs of the chest, when diseased, may carry a morbid action into those of the abdomen; but the modes in which they effect this, are fewer than those in which the latter influence the former.

1. When the heart or lungs is diseased, the digestive organs are liable to suffer from sympathy; of which we have examples in the anorexia and constipation, generally, attendant on acute pneumonic inflammation; in the vomiting which accompanies pericarditis; and, perhaps, in the diarrhœa and fistula in ano, which occur in Phthisis.

2. When, from any cause, the heart is greatly excited, it may raise an inflammation in the abdominal viscera as well as in any other part of the system.

3. When the lungs are so diseased as to perform their function of æreation imperfectly, it is probable, that the matters which they should eliminate, are sometimes thrown upon the liver, or the gastro-enteritic mucus membrane, contributing to the production of diarrhœa.

4, and lastly. When the lungs fail in their transmitting power, or the right side of the heart, which maintains the pulmonary circulation, has suffered such a lesion that it cannot act efficiently, the returning blood is obstructed and accumulated in the venæ cavæ, and the circulation in the liver is of course disturbed. Hence, in part, the hepatic disorders so often present in organic affections of the chest; and so frequently mistaken for the cause of that of which, in reality, they are the effect.

From this synoptic view of the reciprocal morbid influences of the abdominal and thoracic viscera, we might infer that the former preponderate over the latter; in other words, that the heart and lungs are oftener thrown into disorder, by diseases of the liver, stomach and bowels, than the latter are by the maladies of the former; and such we know to be the fact. Thus, observation confirms the suggestions of theory; and demonstrates the utility of a reference to the anatomical and physiological relations of the organs, when we would understand the propagation of morbid actions through the system.

As the object of this little paper was chiefly to indicate the principal modes of morbid intercourse between the organs of two great cavities, I might here bring it to a close; but not having trespassed long upon the patience of the reader, I shall devote a few pages to the morbid influences of the principal viscera of each of those cavities, upon one another.

III. Of the abdominal organs. These are intimately united, both in structure and function. The peritoneum envelopes the whole—their veins unite to form the vena portæ—and their nerves are derived from the great solar plexus. Each organ is destined to perform an office, which is an element in the aggregated function of digestion, and should it fail, the whole abdominal economy is liable to disturbance. Of the modes in which this disturbance may occur, the following are the principal:

1. These organs influence each by sympathy. Thus, acidity of the stomach impairs the peristaltic action of the bowels and *vice versa*. Diseases of the liver produce an irritable stomach, and even excite vomiting. Peritonitis occasions obstinate costiveness. The sympathies of these viscera are, in short, both intense and numerous, as might be inferred from the intimacy of their nervous connexions.

2. When the liver fails in transmitting the blood of the vena portæ, the circulation and specific functions of all the other portal viscera, are necessarily disordered. Among other maladies consequent upon this abdominal plethora, is ascites from increased serous exhalation.

3. When that organ fails to secrete or to excrete bile, the bowels can neither form healthy chyle, nor properly discharge their fœcal contents.

4. When the liver is perverted in its action, or greatly excited, it pours out a biliary fluid, vitiated in quality or redundant in quantity, the stomach and bowels are irritated, and vomiting and diarrhœa, or both, may be the consequence.

5. When the pancreas is enlarged, it sometimes occasions jaundice, by pressure on the common gall duct.

6. When the rectum is contracted, it produces intestinal torpor and remora.

A remark already made, in reference to the reciprocal influence of the thoracic and abdominal organs, is applicable to the morbid influences which the latter exert on each other—it is, perhaps, never in a single mode only. Thus, at the same time, that the diseased liver affects the stomach and bowels, by sympathy—that is, through the medium of the nervous system, producing an altered state of their sensibilities,—it pours into them excessive quantities of acrid bile, which, by its irritation, converts perverted excitability into perverted excitement.

IV. Reciprocal morbid influences of the lungs and heart.

*a.* The lungs, when diseased, disturb the actions of the heart—

1. By sympathy. These great organs are not more remarkable for juxta position, than harmony of action—a relation which could only be established and maintained by the agency of the nervous system. Hence the nerves of both organs are derived from a common plexus, and if either is disturbed in its sensibilities, the other, of necessity, sympathizes. An augmented action of the heart is, therefore, an invariable effect of inordinate pulmonary excitement.

But it is not through the instrumentality of their vital properties only, that diseases of the lungs occasion diseases of the heart. They operate in other modes which are more mechanical, and therefore, more obvious.

2. When, from any cause, the lungs fail properly to æreate the blood, the cavities of the left heart are filled with blood of a bad quality, which must, necessarily, disturb its healthy actions; but the greatest perverted influence is, perhaps, to be found in the admission of this kind of blood into the substance of the heart, through the coronary arteries.

3. When the transmitting power of the lungs is impaired, the blood is necessarily accumulated in the right side of the heart and in the coronary veins, thus producing distension of its cavities and plethora of the vessels of its substance, with great consequent embarrassment of action.

4. In this state of coronary or cardiac plethora, increased serous exhalation may take place, from the internal surface of the pericardium, producing hydropsericardii.

*b*. Diseases of the heart may excite diseases of the lungs in several ways.

1. By sympathy, on which I shall make no remarks.

2. By hypertrophy of the left ventricle diminishing its cavity, or ossification of the mitral or semilunar valves, interrupting the free admission of blood into and out of that cavity; all of which must, necessarily, embarrass the lungs, by impeding the transmission of blood through them.

3. By passive aneurism of the right ventricle of the heart, which being thus weakened, cannot send the blood into the pulmonary artery with sufficient force to secure its proper transmission through the lungs.

4. By active aneurism of the same ventricle, which gives it excess of power over the lungs, and may lead to pulmonary apoplexy, and other forms of sanguineous engorgement.

5. By enlargement of all the cavities of the heart, causing that organ to occupy more than its proper space, and, consequently, preventing the free expansion of the lungs, thus producing dyspnœa.

As in the case of the other organs, the heart seldom, perhaps, exerts itself, unfavourably, on the lungs, in one of these modes only. They are generally combined, though one member of the combination may far exceed the rest in efficiency.

I shall close with a few brief remarks. The human body is a piece of most complicated mechanism, and the assumption, that morbid actions are propagated through it, by any single principle, such as sympathy, or a *vis medicatrix*, must, forever, as it has done heretofore, prove fatal to the system of pathology into which it may be introduced. In every complex machine, *if* one of the component parts should fail in its office, some other will, of necessity, experience injury, and being crippled in its movements, may, ever, disturb the motion of several, until the irritation, by a kind of geometrical progression, is communicated to the whole. But in the living body, long before this takes place, from dependence of function, the parts most remote from that first injured, may be affected, and the whole system involved, in two other modes, examples of which I have already given; but may here refer to them again. Two tissues, each continuous with itself, pervade every part of the body—they are the nervous and the vascular. Of the nature of the agent which resides in the former, we know nothing; but observation teaches us that an impression made on a part, even the most circumscribed, provided the nerves leading to it are perfect, can be felt, in its effects throughout the whole system, even to the annihilation of its vital properties; and, that this phenomena is referable to the nervous system, is apparent, from the fact, that plants which are without a nervous system, at least any distinct tissue of that kind, cannot be *generally* affected by topical applications, and hence their diseases are mostly local and circumscribed.

The other universal tissue has an unquestionable agency in the production of general disease, although by many fashionable writers of the day, its influence is overlooked. Whoever has attentively examined the blood of a patient labouring under an inflammation, must have seen, even while the orifice was still bleeding, that it is by no means in a healthy state. A slate colored surface often begins to appear, at the very moment of its reception in the bowl, indicating, conclusively, that a decomposition into its proximate elements, had begun while it was still in the vessels and circulating through the organs. This, however, is but one of the changes, which it may have undergone, and inflammation is but one form of morbid action. We are, in fact, very far from knowing to what extent and in what manner, the blood may be vitiated in its qualities by the multitude of original morbid states, which may be established in the different organs;—for the colour of the fluid, and the peculiar chemistry which combines its numerous elements, render all researches into its condition exceedingly difficult, and quite uncertain in their results.—Nevertheless, we may be assured of the truth of these two assertions; first, that every local disease must change the qualities of the blood, which is made to circulate through the diseased part: Secondly, that this fluid carried into the other organs, must, necessarily, disturb their vital properties; and hence, that the circulating blood may be the means of awakening diseased organs in parts of the system very distant from that first affected.

(To be continued.)

